# A two-stage temperature control strategy enhances extracellular secretion of recombinant α-cyclodextrin glucosyltransferase in *Escherichia coli*

**DOI:** 10.1186/s13568-017-0465-3

**Published:** 2017-08-23

**Authors:** Yang Li, Jia Liu, Yinglan Wang, Bingjie Liu, Xiaofang Xie, Rui Jia, Caiming Li, Zhaofeng Li

**Affiliations:** 1 State Key Laboratory of Food Science and Technology, Jiangnan University, Wuxi, 214122 People’s Republic of China; 2 School of Food Science and Technology, Jiangnan University, 1800 Lihu Avenue, Wuxi, 214122 Jiangsu People’s Republic of China

**Keywords:** CGTase, Temperature control, Extracellular secretion, Recombinant enzymes, *E. coli*

## Abstract

The effects of temperature on extracellular secretion of the α-cyclodextrin glucosyltransferase (α-CGTase) from *Paenibacillus macerans* JFB05-01 by *Escherichia coli* were investigated. When protein expression was induced at constant temperature, the greatest amount of extracellular recombinant α-CGTase was produced at 25 °C. Higher or lower induction temperatures were not conducive to extracellular secretion of recombinant α-CGTase. To enhance extracellular secretion of α-CGTase by *E. coli*, a two-stage temperature control strategy was adopted. When expression was induced at 25 °C for 32 h, and then the temperature was shifted to 30 °C, the extracellular α-CGTase activity at 90 h was 45% higher than that observed when induction was performed at a constant temperature of 25 °C. Further experiments suggested that raising the induction temperature can benefit the transport of recombinant enzyme and compensate for the decreased rate of recombinant enzyme synthesis during the later stage of expression. This report provides a new method of optimizing the secretory expression of recombinant enzymes by *E. coli*.

## Introduction

The cyclic oligosaccharides α-, β-, and γ-cyclodextrin consist of 6, 7, and 8 glucose units, respectively, linked by α-1, 4-glycosidic bonds. Cyclodextrins form inclusion complexes with many different small, hydrophobic guest molecules, improving their solubility and stability in aqueous environments. This property makes it have many applications in scientific, medical and industrial fields (Roy et al. 2017). The industrial use of α-cyclodextrin is in its infancy, yet is still expanding because of its small internal cavity, high water solubility, and resistance to enzymatic hydrolysis. Previous reports have shown that α-cyclodextrin can be used as a carrier of active ingredients, a solubilizer of lipids, a stabilizer of oils, a modifier of flavors or aromas, and a natural soluble dietary fiber (Aytac and Uyar 2016; Li et al. 2010b, 2014a).

With the expanding use of cyclodextrins on an industrial scale, the cyclodextrin glucosyltransferases (CGTases, EC 2.4.1.19), which catalyze the formation of cyclodextrins, have received increased scientific interest. Although CGTases can be obtained from a wide range of bacteria, the characteristics of the CGTases from *Bacillus* strains are among the closest to industrial requirements (Tonkova 1998). Early work focused on CGTase production in *Bacillus* strains (Gawande et al. 1998; Rosso et al. 2002), and efforts were made to improve CGTase yield by manipulating environmental factors (Arce-Vazquez et al. 2016; Es et al. 2016). Unfortunately, the strict regulatory mechanisms present in wild-type strains have limited productivity enhancements, resulting in high costs and low yields.

A substantial improvement in CGTase expression was observed when the overexpression was performed in recombinant *Escherichia coli* (Mana et al. 2015; Sonnendecker et al. 2017). Unfortunately, previous reports have demonstrated that the CGTases expressed in *E. coli* usually accumulated in the cytosol as biologically inactive inclusion bodies (Makrides 1996; Choi and Lee 2004), and the refolding processes have been proved to be inconvenient (Li et al. 2005). Although secretion into the periplasm is helpful for the rapid isolation of recombinant proteins, current methods for the selective release of periplasmic proteins are not suitable for large-scale production (Yang et al. 1998; Jeang et al. 2005). Therefore, the limitations of cytosolic and periplasmic expression of CGTase make the extracellular secretion of CGTases highly needed.

In our previous study, the α-CGTase gene from *Paenibacillus macerans* JFB05-01 was cloned into the plasmid vector pET-20b(+). This plasmid was then inserted into *E. coli* BL21(DE3) to form a strain used for the extracellular expression of α-CGTase by *E. coli* (Li et al. 2010a, b). The greatest amount of extracellular recombinant α-CGTase was produced when expression was induced at a constant temperature of 25 °C (Li et al. 2010a, b). Extracellular α-CGTase secretion was inhibited when expression was induced at temperatures >30 °C, and very little recombinant enzyme was obtained at 37 °C (Li et al. 2010a, b). Additional studies were devoted to improving the yields of these recombinant α-CGTase by optimizing the composition of the culture medium (Ding et al. 2010; Li et al. 2013a, b). When a one-stage temperature control strategy was used, the membrane permeability was generally at a low level. This low degree of membrane permeability did not favor the secretion of mature α-CGTase into the culture medium. Using a variable temperature control strategy, the membrane permeability may be increased. Therefore, in this study, a novel two-stage temperature control strategy was developed to further improve extracellular expression of *P. macerans* α-CGTase by *E. coli.* The underlying mechanisms for the enhanced enzyme secretion are discussed.

## Materials and methods

### Expression plasmid and chemicals

Construction of the recombinant plasmid *cgt*/pET-20b(+), which directs expression of the wild-type α-CGTase from *P. macerans* strain JFB05-01 (CCTCC M203062) fused to the *pel*B signal peptide, has been described in a previous report (Li et al. 2009). Peptone and yeast extract powder were obtained from Oxoid (Basingstoke, Hampshire, United Kingdom). Isopropyl β-d-1-thiogalactopyranoside (IPTG), *O*-nitrophenyl-β-d-galactopyranoside (ONPG) and *N*-phenyl-α-naphthylamine (NPN) were purchased from Beyotime Institute of Biotechnology (Nantong, China). Glycerin and methyl orange were purchased from Shanghai Chemical Reagent Ltd. (Shanghai, China). All inorganic compounds were of reagent grade or higher quality.

### α-CGTase expression

A single colony of *E. coli* BL21 (DE3) harboring plasmid *cgt*/pET-20b(+) was used to inoculate 50 mL of Luria–Bertani (LB) medium supplemented with 100 mg/mL ampicillin (inoculum size, approximately 0.1%). This starter culture was incubated on a rotary shaker (200 rpm) at 37 °C until the optical density at 600 nm (OD_600_) reached 0.6 (about 8 h). The resulting culture was diluted (1:25) into 100 mL of terrific broth medium in a 500-mL flask, and IPTG was added to a final concentration of 0.01 mM to induce protein expression. The induction was allowed to proceed on a rotary shaker (200 rpm) at the specified temperature for 90 h. Samples of the culture were taken at intervals and analyzed for cell concentration and enzyme activity.

### Cell fractionation

Cell fractionation was performed as previously described, with minor modifications (Li et al. 2010a, b). A 1-mL sample of the culture solution was centrifuged at 10,000 rpm for 10 min and the supernatant was collected. To separate the periplasmic fraction, the bacterial pellet from the 1-mL sample was washed twice with pure water and then completely resuspended in pure water containing 25% (w/v) sucrose and 1 mM EDTA. This suspension was adjusted to a final volume of 1 L, incubated on ice for 2 h, and then centrifuged at 10,000 rpm for 5 min. The supernatant was collected as the periplasmic fraction. The pellet was resuspended in l mL of 10 mM sodium phosphate buffer (pH 6.2) containing 0.5 mM calcium chloride and disrupted by ultrasonication with a sonifier (Branson, USA) for 5 min. After centrifugation at 10,000 rpm for 10 min, the residual cell fragments were mixed with 100 μL of 1% (w/v) SDS-PAGE loading buffer and heated for 10 min in a boiling water bath. After a final centrifugation, the α-CGTase inclusion bodies were in the upper buffer.

### α-CGTase activity assay

α-CGTase activity was determined using the methyl orange method (Li et al. 2013a, b). The culture supernatant (0.1 mL) was mixed with 0.9 mL of 5% (w/v) soluble starch in 50 mM phosphate buffer (pH 6.0) and incubated at 40 °C for 10 min. After terminating the reaction by the addition of 1.0 mL HCl (1.0 M), 1.0 mL of 0.1 mM methyl orange in 50 mM phosphate buffer (pH 6.0) was added. After the mixture had reacted at 16 °C for 20 min, the amount of α-cyclodextrin in the mixture was determined by measuring the absorbance at 505 nm. One unit of α-cyclodextrin-forming activity was defined as the amount of enzyme able to produce 1 µmol of cyclodextrin per min.

### Analysis of inner and outer membrane permeability

Samples were removed from the fermentation specified times after induction and centrifuged at 10,000 rpm for 10 min. The cell pellets were washed twice with 10 mM sodium phosphate buffer (pH 7.4) and diluted with the same buffer until the OD_600_ reached 0.5. Samples (1 mL) of these cell suspensions were used to assess the permeability of their inner and outer membranes as described below.

A previously described absorbance assay was used to evaluate the permeability of the inner membrane (Liao et al. 2004). Briefly, cell samples described above were mixed with ONPG (100 μg/mL) to assess permeability of the inner membrane. Cleavage of the ONPG that entered the cell, which is catalyzed by cytosolic β-galactosidase, was determined by measuring the absorption of light at 420 nm using a spectrophotometer. Measurements were taken every 5 or 10 min for 2 h.

Completeness of the outer membrane was assessed using a previously described NPN fluorescence assay (Eriksson et al. 2002). The fluorescence of NPN is weaker in aqueous solution than it is in hydrophobic environments. When NPN is applied to intact cells, it is excluded from the cells’ interior by the lipopolysaccharide layer of the cells’ outer membranes. Once the outer membrane is compromised, NPN gains access to the lipid bilayer and its fluorescence becomes strong in this hydrophobic environment. Cell suspensions (1 mL, prepared as described in the previous paragraph) were treated with NPN at a final concentration of 10 mM. Fluorescence was measured every 5 or 10 min for 2 h using excitation and emission wavelengths of 350 and 428 nm, respectively, and slit widths of 1 nm. Elevated NPN fluorescence was considered evidence of compromised cell membranes.

### Statistical analysis

All measurements were performed in triplicate. The mean and standard deviations of the data collected were calculated using SPSS 17.0 software (SPSS Incorporated, Chicago, Illinois, USA).

## Results

### A two-stage temperature control strategy enhanced extracellular α-CGTase secretion

**Fig. 1:**
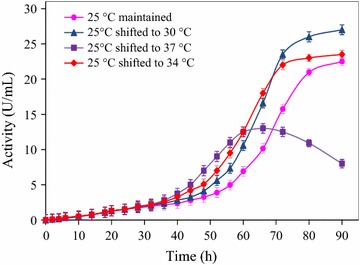
Effect of second-stage temperature on extracellular α-CGTase production. After α-CGTase expression was induced at 25 °C for 24 h, the temperature was shifted directly to 30 °C (▲), 34 °C (♦), or 37 °C (■). The temperature of the control culture (●) was maintained at 25 °C

In an effort to enhance extracellular α-CGTase expression, a novel two-stage temperature control strategy, incubating the expression strain at 25 °C for 24 h and then raising the temperature directly to 30, 34, or 37 °C was investigated. The two-stage temperature control strategy had substantial effects on extracellular α-CGTase production (Fig. 1). A control culture maintained at 25 °C showed a slow rise in extracellular α-CGTase activity until about 50 h of cultivation, a rapid rise between 50 and 80 h of cultivation, then a leveling off between 80 and 90 h of cultivation. Shifting the temperature to 30 °C at 24 h accelerated the increase in extracellular α-CGTase activity between 24 and 80 h of cultivation, compared with the control. At 90 h of cultivation, the enzyme activity reached approximately 27 U/mL, which was 1.2 times that of the control. Shifting the temperature to 34 °C at 24 h further accelerated the increase in extracellular α-CGTase activity when comparing with the increase seen at 30 °C, but in this culture, the plateau was reached earlier (approximately 70 h of cultivation) and the activity at 90 h was not a significant improvement over the level seen in the control culture. Shifting the temperature to 37 °C at 24 h gave the greatest initial rise in extracellular α-CGTase activity; however, the activity peaked at a low activity level at 65 h, and then decreased.

**Fig. 2:**
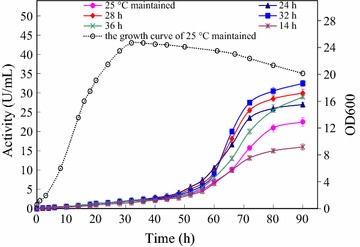
Effect of the temperature change time on extracellular α-CGTase production. Induction of α-CGTase expression was initiated at 25 °C. The induction temperature was shifted directly to 30 °C after 14 h (*), 24 h (▲), 28 h (♦), 32 h (■) or 36 h (×), of induction. The temperature of the control culture (●) was maintained at 25 °C. The growth curve of the control culture is shown as a *dashed line*

Encouraged by the modest increase in extracellular α-CGTase activity observed when the temperature shift occurred at 24 h of cultivation, the temperature shift from 25 to 30 °C was conducted at 14, 24, 28, 32, or 36 h of cultivation (Fig. 2). Shifting the temperature during mid-log growth (14 h) gave the poorest extracellular α-CGTase activity at 90 h of cultivation. Results improved as the timing of the temperature was delayed until a maximum was reached when the temperature was shifted at 32 h of cultivation (late logarithmic growth). Shifting the temperature at the onset of stationary phase (36 h of cultivation) gave substantially poorer extracellular α-CGTase activity at 90 h of cultivation. When the temperature was changed from 25 to 30 °C at 32 h and fermentation was allowed to continue for a total of 90 h, the enzyme activity reached 32.5 U/mL, which was 1.45 times that of the control.

### Cell membrane permeability increased with time and temperature

**Fig. 3:**
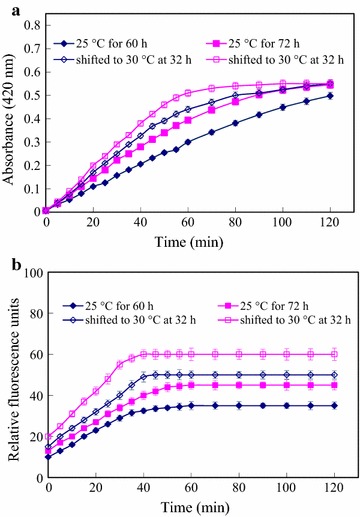
Permeability of *E. coli* cell membranes after induction at different temperatures and for different lengths of time. **a** Outer membrane permeability was assessed using NPN fluorescence after expression had been induced at 25 °C for 60 h (♦) and 72 h (■), or at 25–30 °C (shifting from 25 °C to 30 °C at 32 h) for 60 h (◊) and 72 h (□), respectively. **b** Inner membrane permeability was assessed using ONPG absorbance after expression had been induced at 25 °C for 60 h (♦) and 72 h (■), or at 25–30 °C (shifting from 25 to 30 °C at 32 h) for 60 h (◊) and 72 h (□), respectively. Increases in NPN fluorescence and ONPG absorbance reflect increases in membrane permeability

To investigate the mechanisms enhancing extracellular expression, the permeability of the *E. coli* outer membrane was assessed using the fluorescent probe NPN (Fig. 3a). At the same time, the permeability of the inner membrane was assessed using the colorimetric probe ONPG (Fig. 3b). The permeabilities of both membranes increased with induction time (Fig. 3). Furthermore, the induction temperature shift from 25 to 30 °C resulted in the obvious increase in the permeabilities of both membranes (Fig. 3).

### Movement of α-CGTase between compartments

**Fig. 4:**
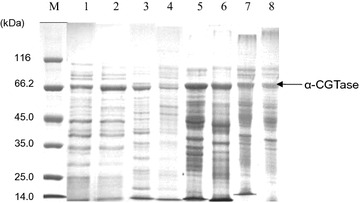
SDS-PAGE analysis of recombinant α-CGTase localization in *E. coli*. *Escherichia coli* cells had been induced at a constant 25 °C for 90 h (*lanes 1*, *3*, *5*, *7*) or using the two-stage temperature control strategy of 25 °C for 32 h followed by 30 °C for 58 h (*lanes 2*, *4*, *6*, *8*). *M* molecular weight standards, *lanes 1* and *2* extracellular fractions, *lanes 3* and *4* periplasmic fractions, *lanes 5* and *6* insoluble inclusion bodies, *lanes 7* and *8* soluble cytopiasmic fraction

To further investigate the mechanisms enhancing extracellular expression, the SDS-PAGE was used to show the amount of α-CGTase protein in the medium, the periplasmic space, the cytoplasm (as a soluble protein) and the insoluble inclusion bodies. The results using the control strategy (induction at 25 °C for 90 h) and the one using the two-phase induction strategy (induction at 25 °C for 32 h, then induction at 30 °C for an additional 58 h) were compared in Fig. 4. The two-phase induction strategy reduced the amount of protein in the periplasmic fraction (lane 4 versus lane 3) and the insoluble inclusion bodies (lane 6 versus lane 5) while it increased the amount of α-CGTase in the medium (lane 2 versus lane 1).

**Fig. 5:**
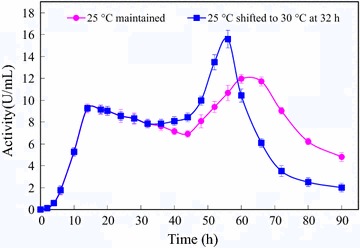
Effect of the fermentation temperature profile on α-CGTase activity in the periplasmic space. Induction temperature was maintained at 25 °C (●), or performed using the two-stage temperature profile beginning at 25 °C and shifting to 30 °C at 32 h (■)

To investigate the time course of α-CGTase movement, the activity of the α-CGTase in the periplasm was determined as a function of time (Fig. 5). Periplasmic α-CGTase activity increased dramatically during the first 15 h of induction at 25 °C, and then had a slow decline. When maintained at 25 °C, the decline continued until 45 h, and then there was a slow increase followed by a slow decline. This produced a peak of periplasmic activity at approximately 65 h of induction. Shifting the induction temperature to 30 °C after 32 h of induction at 25 °C increased the periplasmic α-CGTase activity substantially until it peaked at approximately 55 h. Thereafter, the activity dropped dramatically until, at 90 h, the activity was much lower than that observed when the induction temperature was maintained at 25 °C. These data are consistent with the SDS-PAGE study, suggesting that the two-phase induction strategy increased the flux of α-CGTase through the periplasmic space.

## Discussion

### High initial induction temperature hinders the α-CGTase translocation

Extracellular α-CGTase production occurs in a series of steps. The pre-α-CGTase produced on the ribosome contains an N-terminal *pelB* signal peptide sequence. This N-terminal signal peptide directs translocation of the pre-α-CGTase across the inner membrane to the periplasmic space via the *SecB* pathway (Su et al. 2012). During this process, the signal peptide is removed. Once α-CGTase enters the periplasm, it has two potential fates: it can pass through the outer membrane and enter into the culture medium, or it can aggregate and form inclusion bodies in the periplasmic space (Li et al. 2014a, b).

A previous study showed that the greatest amount of extracellular α-CGTase was produced when the induction was conducted at a constant temperature of 25 °C (Li et al. 2010a, b). Extracellular α-CGTase production was inhibited when the induction temperatures was >30 °C, and very little recombinant enzyme could be obtained at 37 °C. The reason of this phenomenon was probably that the pre-α-CGTase formed inclusion bodies at the inner membrane at temperature above 30 °C, which could block the pre-protein translocation channels and suppress the entrance of newly synthesized pre-CGTase into the periplasm (Chen et al. 2014). At 25 °C, pre-α-CGTase synthesis proceeded at a desirable rate and most of the pre-CGTase passed smoothly through the inner membrane into the periplasm, and then folded correctly (Mergulhao et al. 2005; Fang et al. 2010). The rate of protein synthesis at 25 °C may have prevented the target protein from saturating the secretion machinery and have facilitated the translocation of α-CGTase across both *E. coli* membranes (Yamabhai et al. 2008; Fang et al. 2010).

### A two-stage temperature control strategy enhanced the extracellular α-CGTase production

Having previously established that an initial induction temperature of 25 °C was optimal (Li et al. 2010a, b), we considered changes that would increase α-CGTase flux across both the inner and outer membranes without also increasing the rate of α-CGTase aggregation in the cytoplasm or periplasmic space. Increasing the temperature is a reasonable strategy to increase the α-CGTase movement across the membranes since it can increase the membrane permeability. However, prolonged induction periods at elevated temperature, especially at 37 °C, may cause cell lysis, which would decrease productivity and secretion capacity (Mana et al. 2015). Therefore, the second-stage temperatures between 30 and 37 °C were investigated during the total induction period of 90 h. The timing of the temperature shift had to be selected to minimize formation of inclusion bodies through accelerating translation. In the first experiment, 24 h was chosen because the inspection of the growth curve (Fig. 3) revealed that 24 h was well past the mid-point. The results showed that increasing the temperature increased the initial rate of α-CGTase production, but temperatures >30 °C gave poorer overall yields at 90 h of induction. This phenomenon was perhaps due to the premature cell lysis (Mana et al. 2015).

After selecting 30 °C as the optimal second-stage temperature, we decided to further investigate the timing of the temperature shift. The time course of α-CGTase activity in the periplasmic space during induction at 25 °C was clearly biphasic, with an early peak at 14 h of induction and a late peak at approximately 64 h (Fig. 5). We decided to investigate the temperature shift times beginning with the early peak (14 h, Fig. 5) and extending through late-log phase (36 h, Fig. 3). The optimal yield of the extracellular α-CGTase was finally obtained with a temperature shift time of 32 h (Fig. 5).

### Enhanced membrane permeability increased α-CGTase production

The mechanistic data presented in Figs. 3, 4 and 5 strongly suggest that the two-stage induction strategy increased membrane permeability, which caused increased extracellular α-CGTase production. The increased inner membrane permeability in the ONPG study (Fig. 3) and the decreased amount of cytosolic protein and inclusion bodies in the SDS-PAGE study (Fig. 4) suggest that the two-stage induction strategy increased *E. coli* inner membrane permeability and then accelerated the transit of the α-CGTase across the inner membrane. This is confirmed by the increased α-CGTase activity that observed in the periplasmic space between 32 and 60 h of induction (Fig. 5). This accumulation of the α-CGTase activity in the periplasmic space further suggests that outer membrane permeability plays a significant role in the extracellular expression. The low degree of the outer membrane permeability was the main reason why only a small portion of the mature α-CGTase was secreted into the culture medium during the early stage of induction. The increased permeability of the outer membrane in the NPN study (Fig. 3) and the decreased amount of periplasmic α-CGTase in the SDS-PAGE study (Fig. 4) suggest that the two-stage induction strategy increased the *E. coli* outer membrane permeability, and then accelerated the transport of the α-CGTase across the outer membrane. This is confirmed by both the rapid decrease in the periplasmic α-CGTase activity during 60–90 h of induction and the simultaneous increase in the extracellular α-CGTase activity (Fig. 2).

In summary, this study showed that when the α-CGTase from *P. macerans* strain JFB05-01 was expressed in *E. coli* as a recombinant fusion protein carrying a *pel*B leader sequence, a two-stage induction temperature control strategy can help to obtain the optimal extracellular α-CGTase production. In this two-stage induction temperature control strategy, induction was conducted at 25 °C for 32 h, and then the temperature was shifted to 30 °C and the induction was continued for an additional 58 h. Using this two-stage induction control strategy, the extracellular α-CGTase activity was increased by 45% compared with the induction at a constant temperature of 25 °C. The primary mechanism responsible for the increase of the α-CGTase production was due to the increase of the membrane permeability. This is the first report describing a two-stage temperature control strategy used for increasing the extracellular α-CGTase production in *E. coli*.

## Abbreviations

α-CGTase: α-cyclodextrin glucosyltransferase
*E. coli*: *Escherichia coli*
IPTG: isopropyl β-d-1-thiogalactopyranoside
ONPG: *O*-nitrophenyl-β-d-galactopyranoside
NPN: *N*-phenyl-α-naphthylamine
LB: Luria–Bertani
OD: optical density
EDTA: ethylenediaminetetraacetic acid
SDS-PAGE: sodium dodecyl sulfate polyacrylamide gel electrophoresis
